# Formamide as the main building block in the origin of nucleic acids

**DOI:** 10.1186/1471-2148-7-S2-S1

**Published:** 2007-08-16

**Authors:** Giovanna Costanzo, Raffaele Saladino, Claudia Crestini, Fabiana Ciciriello, Ernesto Di Mauro

**Affiliations:** 1 Istituto di Biologia e Patologia Molecolari, CNR, P.le Aldo Moro, 5, Rome 00185, Italy; 2 Dipartimento A.B.A.C., Università della Tuscia, Via San Camillo De Lellis, Viterbo, Italy; 3 Marine Dipartimento di Scienze e Tecnologie Chimiche, Università "Tor Vergata", Rome 00133, Italy; 4 Fondazione "Istituto Pasteur-Fondazione Cenci-Bolognetti" c/o Dipartimento di Genetica e Biologia Molecolare, Università "La Sapienza" di Roma, P.le Aldo Moro, 5, Rome 00185, Italy; 5 Ernesto Di Mauro, Dipartimento di Genetica e Biologia Molecolare, Università "La Sapienza" di Roma, P.le Aldo Moro, 5, 00185 Rome, Italy

## Abstract

The simplest molecules grouping the four most common elements of the universe H,C,O and N (with the exception of the biologically inert He) are isocyanate HNCO and formamide H_2_NCOH. Reasons for the availability of formamide on prebiotic Earth are presented. We review evidence showing that formamide in the presence of largely available catalysts and by moderate heating yields the complete set of nucleic bases necessary for the formation of nucleic acids. Formamide also favours the formation of acyclonucleosides and the phosphorylation and trans-phosphorylation of nucleosides, thus providing a plausible chemical frame for the passage from a simple one-carbon compound to nucleic polymers. Physico-chemical conditions exist in which formamide favours the stability of the phosphoester bonds in nucleic polymers relative to that of the same bonds in monomers. Starting from a formamide-laden environment subject only to the laws of chemistry, a hypothesis is outlined sketching the passage towards an aqueous world in which Darwinian rules apply.

## Background

Life is a sturdy phenomenon and its initial steps *bona fide *originated from robust chemical frames based on firm thermodynamic ground. These assumptions on the simplicity and the necessity of the pre-biogenic processes are mitigated by the consideration that the genetic mechanisms onto which relies life-as-we-know-it today are combinatorially elaborated. In passing from the initial self-organization of chemical information to the potentially infinite complexity of interplaying genotypes and phenotypes that we experience today, evolution did necessarily play the key role.

We have focused on two aspects of the problem: the definition of a plausible chemical frame into which the first spontaneous syntheses could have taken place; the evolutionarily relevant selective properties and constrains that the first informational polymers had to deal with. The two aspects are intimately connected.

## Results

### Is formamide a plausible prebiotic precursor?

The nature of the chemicals that played the role of prebiotic precursors on primitive Earth is still a debated argument. In a general approach to the problem, the following physical and chemical properties of the simple organic compounds under consideration should be taken into account. Namely: (i) the relative abundance of the starting biogenic materials, to be considered a pre-requisite for the early onset [1-4] of genetic processes on this planet; (ii) their stability; (iii) their ability to react to give more complex structures following reproducible pathways. The formation of precursors based on simple chemical processes, and the *quasi*-simultaneous presence of all the building blocks to be used for the assembling of informational molecules, are other important requisites.

#### (i) Availability

Formamide (H_2_NCOH) meets the required criteria of abundance and diffusion in the Universe. The analysis of the molecular composition of comets-asteroids and of the interstellar clouds shows that the compounds made of the 4 more common and biologically relevant elements H, O, C and N (excluding He) are isocyanate HNCO and formamide H_2_NCOH [5]. Formamide was detected in the gas phase of interstellar medium [6], in the long period comet Hale-Bopp [7], and tentatively in the solid phase of grains around the young stellar object W33A [8]. Possible formamide production under *Europa*-like conditions was observed (Hand, K.; Carlson, R. W., Department of Geological & Environmental Sciences, Stanford University; personal communication, July 2006).

#### (ii) Stability

**Figure 1:**
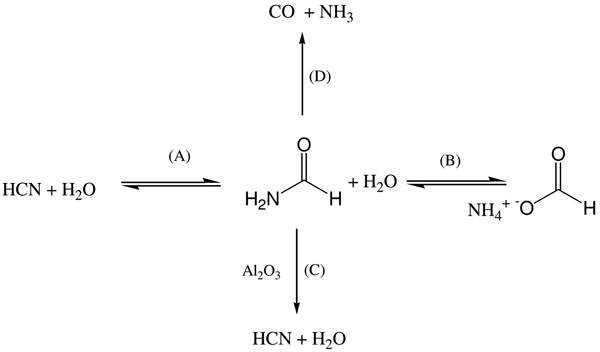
**The basic formamide chemistry**. A scheme summarizing basic formamide chemistry; more details in text.

Formamide meets the required criteria of stability. This topic must be considered in connection with hydrogen cyanide (HCN) chemistry. Since the pivotal experiment by Orò [9] on the synthesis of adenine from HCN, numerous studies were devoted to assess the role of this compound in the origin of primordial nucleic acids [10]. Nevertheless, two problems remain unsolved in the prebiotic relevance of HCN chemistry: (i) the thermodynamic instability of HCN under hydrolytic conditions, (ii) the narrow panel of nucleobases, limited only to purines that can be formed by its condensation process. In the perspective of this latter observation, an all-purine precursor of nucleic acids was proposed, in which the pyrimidines present in extant nucleic acids would be post-enzymatic substitutes for their isoelectronic and isogeometric purines [11]. HCN is a gas under a wide range of environmental conditions. Thus, HCN chemistry in homogeneous solution (the largely accepted chemical prebiotic *scenario *on the primitive Earth) firstly requires absorption in water. After the adsorption process polymerization and hydrolysis of HCN compete, the results being determined by its concentration. The two reactions are equivalent at concentrations of HCN between 0.01 and 0.1 M (between pH 8 and 9). Hydrolysis to formamide (Figure 1, equation A) predominates in dilute solutions while polymerization takes over at higher concentrations [12]. The steady state concentration of HCN in the primitive ocean was calculated, on the basis of the estimated rates of its production and hydrolysis, to be at pH7 4 × 10^-12 ^M at 100°C and 2 × 10^-5 ^M at O°C. These concentrations are far too low for polymerization to nucleobases to occur, thus favouring hydrolysis to formamide [13,14].

Since HCN is more volatile than water it cannot be concentrated by simple evaporation at pH lower than its pKa (9.2 at 25°C)[15]. This suggested eutectic freezing as a means for HCN to reach the sufficient concentration for polymerization [16].

In the same study, the hydrolysis rate (and the steady state concentration) of formamide to ammonium formate (Figure 1, equation B) was also estimated as 2 × 10^-18^, 1 × 10^-15 ^and 1 × 10^-9 ^M, at 200, 100 and 0°C respectively, assuming that in the primitive ocean formamide was formed only by HCN hydrolysis.

On the basis of these data the authors suggest that "it is unlikely that formamide could have served a significant role in the prebiotic chemistry", a quite definitive sentence for this compound!

However, this assumption does not take into consideration that (i) formamide can be formed from prebiotic compounds largely diffused on the primitive Earth other than HCN, and (ii) that formamide is liquid under a wide range of temperature and pressure values, with a boiling point of 210°C and very limited azeotropic effects [17]. Thus, unlike HCN, formamide in a drying lagoon model can be easily concentrated, increasing its stability upon concentration and providing the adequate concentration for polymerization to nucleobases to occur. The hydrolysis of formamide in water was revisited by studying the solvent deuterium kinetic isotope effect. This analysis provided a value of the constant k_hyd _of 1.1 × 10^-10 ^s^-1^, corresponding to a t_1/2 _of ca. 200 yr at 25°C and pH 7.0 [18].

#### (iii) Reactivity

As an organic compound able to generate "*in situ*" many other simple chemicals useful for the synthesis of nucleobases, formamide can be considered as a multifunctional prebiotic precursor. The ratio of the afforded precursors depends on the specific environmental conditions.

At 190–210°C under atmospheric pressure formamide thermally decomposes either to ammonia (NH_3_) and carbon monoxide (CO) (Figure 1, equation C) or to HCN and water (Figure 1, equation D). The formation of HCN is usually favoured in the presence of suitable catalysts, i.e., with aluminium oxides the yield at temperatures between 400°C and 600°C is >90%, while in the absence of catalysts the reaction forming NH_3 _and CO predominates [19]. Further decomposition products are also detected. These include polymeric hydrogen cyanide derivatives [20] potentially producing nucleobases under hydrolytic conditions. Due to its high dielectric constant [21] formamide is in addition an excellent solvent for both metal oxides and inorganic salts, which can act as catalysts during the condensation processes to nucleobases.

Thus, the composition of a reaction mixture based on formamide as the main component is tuned by the composition of the environmental reactor providing, at difference from HCN, all the prebiotic precursors necessary for the synthesis of both purine and pyrimidine nucleobases. The composition of the panel of the prevailing products depends on the specific physical and chemical properties of the catalysts present in the reaction medium, as detailed below.

### The synthesis of nucleic precursors from formamide

#### Nucleic bases, one amino acid and a condensing agent

##### Nucleic bases

We have observed that formamide has the unique property of condensing into both purine and pyrimidine nucleobases simply upon heating at 110–160°C in the presence of largely diffused metal oxides and minerals [22-26]. The products obtained are listed in Table 1, crossed with the catalysts tested and grouped as a function of (approximate) increasing complexity. Purine is the only compound obtained by heating formamide in the absence of catalysts. The most relevant aspects of this large ensemble of products are:

**Table 1:** Catalysed synthesis of nucleic acid components and precursors from formamide

				**Pyrimidines**							**Imidazoles**			**Purines**				
			
**Catalyst\Product**	1	2	3	4	5	6	7	8	9	10	11	12	13	14	15	16	17	18
Silica	**-**	**-**	**-**	**+**	**-**	**-**	**-**	**-**	**+**	**-**	**-**	**-**	**-**	**+**	**-**	**+**	**-**	**-**
Alumina	**-**	**-**	**-**	**+**	**-**	**-**	**-**	**-**	**+**	**-**	**-**	**-**	**-**	**+++++**	**-**	**+**	**-**	**-**
Kaolin	**-**	**-**	**-**	**+**	**-**	**-**	**-**	**-**	**-**	**-**	**-**	**-**	**-**	**+++++**	**-**	**-**	**-**	**-**
Zeolite	**-**	**-**	**-**	**+**	**-**	**-**	**-**	**-**	**+**	**-**	**-**	**-**	**-**	**+++++**	**-**	**+**	**-**	**-**
CaCO_3_	**-**	**-**	**-**	**-**	**-**	**-**	**-**	**-**	**-**	**-**	**-**	**-**	**-**	**+++++**	**-**	**-**	**-**	**-**
KP-10 Clay^a^	**-**	**-**	**-**	**-**	**-**	**+**	**-**	**-**	**+++**	**-**	**-**	**-**	**++**	**++++**	**+++**	**++**	**-**	**+**
K-30 Clay^a^	**-**	**-**	**-**	**-**	**-**	**+**	**-**	**-**	**+++**	**-**	**-**	**-**	**++++**	**+**	**++++**	**++++**	**-**	**-**
KSF Clay^a^	**-**	**-**	**-**	**-**	**-**	**+**	**-**	**-**	**+++**	**-**	**-**	**++++**	**+++**	**++++**	**+**	**+++**	**-**	**+**
Al-PILC Clay^a^	**-**	**-**	**-**	**-**	**-**	**+**	**-**	**-**	**+++**	**-**	**-**	**+++**	**+**	**+++**	**+**	**+**	**-**	**+**
TiO_2_	**-**	**-**	**-**	**-**	**-**	**-**	**+**	**-**	**+**	**+**	**-**	**-**	**-**	**+++**	**+++**	**++**	**+**	**-**
MgFeSiO_4_	**+**	**-**	**-**	**-**	**++**	**+**	**-**	**-**	**+++**	**-**	**-**	**-**	**-**	**+**	**-**	**-**	**-**	**-**
Mg_1.5_Fe_0.5_SiO_4_	**+**	**-**	**-**	**-**	**++**	**-**	**-**	**-**	**++++**	**-**	**-**	**-**	**-**	**-**	**-**	**-**	**-**	**-**
Mg_0.5_Fe_1.5_SiO_4_	**-**	**-**	**-**	**-**	**+++++**	**+**	**-**	**-**	**+++**	**-**	**-**	**-**	**-**	**-**	**-**	**-**	**-**	**-**
Fe_2_SiO_4_	**+**	**-**	**-**	**-**	**+++++**	**+**	**-**	**-**	**+++++**	**-**	**-**	**-**	**-**	**+**	**-**	**-**	**-**	**-**
Mg_2_SiO_4_	**+**	**-**	**-**	**-**	**-**	**-**	**-**	**-**	**-**	**-**	**-**	**-**	**-**	**+**	**-**	**-**	**-**	**-**
Na_3_PO_4_	**+**	**+**	**++**	**-**	**-**	**+**	**-**	**-**	**++++**	**-**	**-**	**-**	**-**	**+++**	**-**	**-**	**-**	**-**
Na_4_P_2_O_7_	**-**	**+**	**+**	**-**	**-**	**+**	**-**	**+**	**+++**	**-**	**-**	**-**	**-**	**++**	**-**	**+**	**-**	**+**
Na_5_P_3_O_9_	**-**	**+**	**++**	**-**	**-**	**-**	**-**	**-**	**+**	**-**	**-**	**-**	**-**	**+**	**-**	**-**	**-**	**-**
Turquoise^c^	**-**	**-**	**+**	**-**	**-**	**-**	**-**	**-**	**++**	**-**	**-**	**-**	**-**	**++**	**-**	**-**	**-**	**-**
Childrenite^c^	**+**	**-**	**+++++**	**-**	**-**	**-**	**-**	**-**	**-**	**-**	**-**	**-**	**-**	**-**	**-**	**-**	**-**	**-**
Ludlamite^c^	**-**	**-**	**+++++**	**-**	**-**	**-**	**-**	**-**	**-**	**-**	**-**	**-**	**-**	**+**	**-**	**-**	**-**	**-**
Vivianite^c^	**+**	**-**	**++++**	**-**	**-**	**-**	**-**	**-**	**-**	**-**	**-**	**-**	**-**	**+**	**-**	**-**	**-**	**-**
Vauxite^c^	**-**	**-**	**++++**	**-**	**-**	**-**	**-**	**-**	**-**	**-**	**-**	**-**	**-**	**+**	**-**	**-**	**-**	**-**
Lazulite^c^	**-**	**-**	**+++**	**-**	**-**	**+**	**-**	**-**	**+**	**-**	**+**	**-**	**-**	**+**	**-**	**-**	**-**	**-**
Hureaulite^c^	**-**	**+**	**-**	**-**	**-**	**+**	**-**	**-**	**++**	**-**	**-**	**-**	**-**	**+**	**-**	**-**	**-**	**-**
Augelite^c^	**-**	**-**	**+++**	**-**	**-**	**-**	**-**	**-**	**+**	**-**	**-**	**-**	**-**	**+**	**-**	**-**	**-**	**-**
Wavellite^c^	**-**	**-**	**+**	**-**	**-**	**-**	**-**	**-**	**+**	**-**	**-**	**-**	**-**	**+**	**-**	**-**	**-**	**-**
Libethenite^c^	**-**	**+**	**+**	**-**	**-**	**+**	**-**	**-**	**+**	**-**	**-**	**-**	**-**	**-**	**-**	**-**	**-**	**-**
Pyromorphite^c^	**-**	**-**	**-**	**-**	**-**	**-**	**-**	**-**	**+**	**-**	**-**	**-**	**-**	**+**	**-**	**-**	**-**	**-**

^a^: Montmorillonite clays, ^b^: Cosmic Dust Analogues, ^c^: phosphate minerals. Data from [22] for the syntheses catalyzed by silica, alumina, kaolin, zeolites, and CaCO_3_; from [23] for TiO_2; _from [24] for clays and from [25] for olivines; from [26] for the soluble and mineral phosphates, as listed; mg of product per gram of formamide. +: 0.1–5.0 mg; ++: 5–10 mg; +++: 10–20 mg; ++++: 20–40 mg; +++++: >40 mg. Products are: 1 = Urea; 2 = Carbodiimide; 3 = *N*-Formylglycine; 4 = Hydroxypyrimidine; 5 = 4(3H)-Pyrimidinone; 6 = Uracil; 7 = 5-Hydroxymethyluracil; 8 = Dihydroxyuracil; 9 = Cytosine; 10 = Thymine; 11 = Parabanic acid; 12 = AICA: 5-Aminoimidazole-4-carboxamide; 13 = fAICA: 5-Formamidoimidazole-4-carboxamide; 14 = Purine; 15 = N^9^-formylpurine; 16 = Adenine; 17 = N^9^, N^6^-Diformyladenine; 18 = Hypoxanthine.

- the panel of compounds obtained in the presence of each catalyst is 'clean'. Only few products are observed, in certain cases the synthesis being highly specific, as in the case of the phosphate mineral pyromorphite yielding exclusively cytosine or in the case of childrenite yielding almost only *N*-formylglycine. In other instances richer panels of products are obtained, as with pyrophosphate Na_4_P_2_O_7 _yielding (in addition to purine) adenine, hypoxantine (a bioisoster of guanine), uracil, cytosine, *N*-formylglycine and carbodiimide; and with TiO_2 _yielding (in addition to purine) adenine, N^9^-formylpurine, N^9^-N^6^-diformyladenine, cytosine, thymine and 5-hydroxymethyluracil.

- Quite interestingly TiO_2 _also catalyzes the synthesis of purine acyclonucleosides (not reported in Table 1, see Ref 23). This observation is of particular prebiotic relevance because of the known difficulty of building under prebiotic conditions the β-glycosidic bond between separately synthesized nucleobases and sugars [27]. Anyhow, even in the instance of relatively higher complexity mixtures the products profiles keep their character of neatness and do not usually contain degradation products nor additional classes of compounds.

##### Glycine and carbodiimide

The α-amino acid derivative *N*-formylglycine was detected in formamide-based syntheses catalyzed by phosphate-minerals, often accompanied by carbodiimide [26]. The synthesis of carbodiimide, which is an important agent for the condensation of aminoacids into peptides, could be responsible for the formation of formylglycine from *in situ *generated glycine [26], suggesting a role for the formamide-phosphate system in the prebiotic synthesis of peptides.

Intermediates of the synthetic pathways for components of extant nucleic acids are also observed, i.e., 4-aminoimidazole-5-carboxamide (AICA), 4-formylaminoimidazole-5-carboxamide (f-AICA) and 5-hydroxymethyluracil.

The chemical mechanisms onto which all these syntheses are based are described and critically discussed in [28-30].

### The chemomimesis concept as a selector of prebiotic precursors

As mentioned above, the identity of the first prebiotic precursors of nucleic acids is still an argument of debate. On the other hand, the analysis of the mechanism of reaction of simple organic molecules reveals instances in which key intermediates are also produced corresponding to those observed in extant biological pathways. The concept of chemomimesis applies to these correspondence. This term, first introduced by Eschenmoser and Loewenthal in 1992 [31], generally refers to a chemical reaction pathway that can be used as a template for the enzymatic processes that will appear later in evolution, yielding the same final products. This property can in principle distinguish two classes of prebiotic precursors: the precursors that are able to generate a chemomimetic process from those that are not. Formamide chemistry shows interesting cases of chemomimesis.

**Figure 2:**
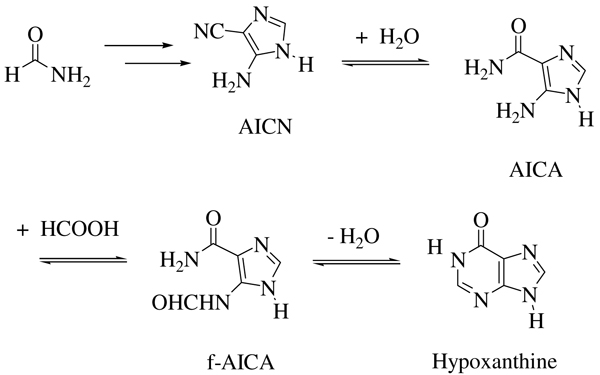
**Chemomimesis instances in formamide chemistry**. See text for details.

As an example, 5-aminoimidazole-4-carboxamide (AICA) and 5-formamido-imidazole-4-carboxamide (f-AICA), obtained in high yield in addition to hypoxanthine upon heating formamide in the presence of montmorillonites (Table 1), are also key intermediates (as ribonucleotide-5'-monophosphates) in the last steps of the extant biosynthesis of inosine-5'-monophosphate (IMP), the main route to purine nucleotides in the cell (Figure 2).

Similarly, the addition of formaldehyde to a preformed uracil scaffold during the synthesis of thymine from formamide and TiO_2 _is a key step for the introduction of the methyl moiety, in agreement with the extant biosynthesis of thymidine. In this reaction a formaldehyde unit is added to uridine, masked as the activated methylene unit of methylene tetrahydrofolate (MTHF) to give 5-hydroxymethyluracil-5'-monophosphate (HMU-5'-monophosphate). Thymidine will be obtained by successive hydride shift rearrangement.

The possibility that early chemical events played the role of templates for the development of more complex (but also more efficient and selective) enzymatic pathways is a fascinating concept to be further evaluated in the study of the molecular evolution of informational polymers.

### Problems in prebiotic polymerization

#### Activated precursors

Evolution of the genetic information based on linear polymers implies a template-mediated replication mechanism. Templated reproduction allows the accumulated information to be maintained and occasionally modified, thus establishing chemically-based evolutionary rules. Non-enzymatic self-replicating systems based on template-directed synthesis of oligonucleotides were reported (among which those reported in [32-39], reviewed in [30]), providing the proof of principle for the plausibility of this general mechanism. The first pre-genetic polymers were not necessarily made of the sugar moieties that compose extant nucleic acids, nor were the nucleosides forcibly connected by the phosphoester linkages that we experience today. Comprehensive analyses of the possible alternatives were reported [10,30,40]. However, in the absence of direct evidences or of solid indications to the contrary, it is safely assumed that genetic evolution originated based on RNA-like polymers, that ribose and phosphodiester bonds were the actual components whose properties allowed and chick-started evolution, that the molecular horses were not changed during the run. The reasons favouring phosphate as a connecting element are well established [41].

A general problem derives from the fact that the formation of a phosphodiester bond is thermodynamically uphill. Thus, the template-directed protein-free prebiotic synthesis of phosphodiester-linked oligonucleotides most probably required the use of chemically activated nucleotides.

#### Formamide-catalyzed phosphorylation of nucleosides

The prebiotic relevance of the mechanisms proposed for the production of activated nucleosides is questionable [10,42]. The otherwise important observation that drying the chemically activated nucleoside 5'-phosphorimidazolide-adenosine (ImpA) onto the surface of a Montmorillonite clay a pre-absorbed decanucleotide could be elongated by up to 30 additional nucleotides [36,37] and similar results in comparable systems in [38], suffer from the same limitation. Thus, an efficient and robust catalytic mechanism for the activation (possibly phosphorylation) of nucleosides was probably involved. We have observed that phosphorylation of nucleosides readily occurs in the presence of formamide and of a phosphate donor. The donor could be a soluble mono-, di- or triphosphate; or a different phosphorylated nucleoside; or one of several crystalline phosphate minerals among which hydroxylapatite, libethenite and pseudomalachite (data not detailed, submitted for publication elsewhere). Phosphorylation occurred on the 5', or the 3', or the 2' C atoms of the ribose moiety, and 2':3' and 3':5' cyclic phosphoester forms were also observed. Based on the ground-breaking observation by Orgel [43] that dinucleoside diphosphates formed from adenosine 2':3' cyclic phosphate, this cyclic phosphate ribonucleotide system is of possible particular prebiotic relevance.

### Aqueous versus non-aqueous

The chemically relatively easy formation of linear polymers from activated precursors does not solve the problem of their origin. The standard-state Gibbs free energy change (ΔG°') problem, as critically evaluated by van Holde [44], and the intrinsic instability of polymers in solution limit the formation and the survival of polymers in aqueous environments. The ΔG°' problem is the major obstacle for liquid-phase polymerizations in prebiotic conditions.

The phosphorylation of nucleosides on mineral surfaces mentioned above was obtained in the presence of formamide. Thus, activated nucleic monomers can form in a liquid *non-aqueous *environment in conditions compatible with the thermodynamics of polymerization, providing an operational solution. If formamide afforded activated precursors by phosphorylation of nucleosides and allowed their polymerization by a simple trans-phosphorylation reaction (a still hypothetical but chemically plausible process), the limiting factor for the evolution of pre-genetic molecules would become the stability of the resulting polymerized forms. In other words, when considering the conditions in which pre-genetic polymers could spontaneously polymerize, replicate and evolve, the physico-chemical parameters favouring or allowing the survival of the very polymeric state are of paramount relevance. Hence the interest of defining the initial thermodynamic niches into which the polymeric state could have been favoured over the monomeric one.

These niches were identified for both the deoxyribo [45] and the ribo [46] systems, showing that defined combinations of temperature and solvent favour the polymeric state. These niches are remarkably broader for RNA than for DNA (cfr data in [45] versus [46]).

In the frame of the "RNA world" hypothesis, this finding shows that in addition to the three important properties of RNA [(i) its capacity to encode and express genetic information; (ii) the variety of structures and hence of functions it assumes; (iii) its catalytic abilities] the property of favoured *persistence *should also be considered. This fourth property consists of the ensemble of thermodynamic and kinetic parameters pertaining to the polymerization process and polymers stability. The conditions favouring oligomeric persistence could potentially provide information about the environment in which the ur-genetic molecules came into existence and survived.

### Stability as critical phenotype for the evolution of informational polymers

In reconstructing the passage from monomers to the information-bearing polymers that we know at present, and summarizing the data reported above, we have observed that formamide 1) condenses into all the nucleic bases necessary to form present-day nucleic acids. This process only requires moderate temperature (110–160°C) and easily available catalysts. 2) Several compounds are formed encasing a hidden β-glycosidic bond, potentially solving the chemical rebus posed by the non-reactivity of nucleic bases with sugars. 3) Formamide-based phosphorylation of preformed nucleosides was observed (data submitted elsewhere), providing a plausible solution to the problem of the chemically robust mechanism needed for a non-fastidious high-yield production of activated precursors. Polymers formation might be hypothesized through a formamide-driven template-directed trans-phosphorylation process. The suitable template could be provided by a mineral surface or by nucleic polymers. These processes provide a simple-chemistry frame into which all the steps from the one-carbon atom compound H_2_NCOH to activated nucleotides have been described. All these reactions require formamide as building material and/or as catalyst.

However, extant organisms live in water, not in formamide. And the structure and properties of nucleic acids strongly hint that interaction with water is one of their most intimate properties. At what stage could the passage from a formamide environment to water have occurred?

At this point we are on hypothetical grounds. Let us start from a model in which the synthesis of a ribo oligonucleotide has occurred by connecting pre-synthesized nucleosides [22–26, especially ref. [23]], phosphorylated by formamide-catalyzed phosphorylation and joined by formamide catalyzed trans-phosphorylation (hypothetical). Phosphodiester bridges between nucleosides could have occurred between monomers bound as single units on phosphate mineral surfaces providing both a source for phosphate moieties and a correct spatial ordering. The P-P distance in a stretched nucleic acid is a well established 9.15 Å, in good correspondence with the crystal cell dimensions of phosphate minerals whose *a *and *b *values in a large number of different minerals are encompassed between 6 and 10 Å [47] (first generation stage). Alternatively, or at a later stage, phosphodiester bridges would have formed between activated monomers bound as single units on nucleic acid template (second generation stage). Both stages require formamide as the driving chemical for phosphate bridge formation.

## Conclusion

Passage to water from a formamide-based chemically active environment may be conceived only if advantageous under Darwinian selection. We have observed [46] that in water the 3' phosphoester bond in RNA (and, remarkably, much less so in DNA) may be more stable that the corresponding bond in the starting monomers. The structural properties conferring such an advantage (i.e., protection towards hydrolysis induced by base stacking, differential deprotonation rates, different kinetics and/or frequency of the cleavage-inducing in-line configuration made of the attacking 2'-oxygen nucleophile with the 5'-oxyanion leaving group and the target phosphorus center [48], etc.) are currently under investigation. Whatever the structural reasons, the higher stability of the polymeric form is exactly what can be seen as an advantageous proto-phenotype, conferring to a neo-formed polymer the ability to maintain the chemical information created by the assembling of dispersed monomers.

In spite of its largely hypothetical character, this model (i) is based on simple state-of-art chemistry, (ii) relies on easily available building material and on common catalysts, (iii) may be brought to experimental verification, and (iv) brings in close proximity a purely chemical world with a system in which properties deriving from combinatorial events become relevant selective attributes. In this overlapping worlds the first parameter for fitness is stability (*first: survive*). Molecules stable enough to reproduce would thus be granted the possibility to explore evolutionary space.

## Competing interests

The authors declare that they have no competing interests.

## Authors' contributions

All authors contributed equally to the experiments and models described.
